# FamAgg: an R package to evaluate familial aggregation of traits in large pedigrees

**DOI:** 10.1093/bioinformatics/btw019

**Published:** 2016-01-22

**Authors:** Johannes Rainer, Daniel Taliun, Yuri D’Elia, Cristian Pattaro, Francisco S. Domingues, Christian X. Weichenberger

**Affiliations:** ^1^Center for Biomedicine, European Academy of Bozen/Bolzano (EURAC) (Affiliated to the University of Lübeck, Lübeck, Germany), Bolzano 39100, Italy and; ^2^Department of Biostatistics and Center for Statistical Genetics, University of Michigan School of Public Health, Ann Arbor, MI 48109-2029, USA

## Abstract

**Summary:** Familial aggregation analysis is the first fundamental step to perform when assessing the extent of genetic background of a disease. However, there is a lack of software to analyze the familial clustering of complex phenotypes in very large pedigrees. Such pedigrees can be utilized to calculate measures that express trait aggregation on both the family and individual level, providing valuable directions in choosing families for detailed follow-up studies. We developed FamAgg, an open source R package that contains both established and novel methods to investigate familial aggregation of traits in large pedigrees. We demonstrate its use and interpretation by analyzing a publicly available cancer dataset with more than 20 000 participants distributed across approximately 400 families.

**Availability and implementation:** The FamAgg package is freely available at the Bioconductor repository, http://www.bioconductor.org/packages/FamAgg.

**Contact:** Christian.Weichenberger@eurac.edu

**Supplementary information:** Supplementary data are available at *Bioinformatics* online.

## 1 Introduction

The investigation of whether a disease or symptom trait recurs more often among close relatives than in the general population is a deeply rooted subject in genetic epidemiology, often termed as familial aggregation analysis (Khoury *et al.*, 1993). While segregation analysis was the tool of choice to identify patterns of Mendelian diseases, there is no unique method to highlight familial clusters for complex diseases, especially in situations involving very large pedigrees lacking a regular family structure. In this setting, more than three decades ago, a computational method was first developed to highlight familial aggregation of various cancer types (Hill, 1980). The method was based on the kinship coefficient Φ*_ij_*, which is the probability that two subjects *i* and *j* share the same allele identical-by-descent at one locus, and represents a suitable measure to quantify the relationship between two individuals in the pedigree (Malécot, 1948). In this early approach, the average kinship between all affected pairs was compared to the mean kinship of multiple sets of randomly selected matched controls (Hill, 1980). This and other kinship-based methods have been successfully applied to very large pedigrees to assess whether diseases such as autism (Jorde *et al.*, 1990) or Parkinson’s disease (Sveinbjörnsdottir *et al.*, 2000) showed evidence of familial aggregation. The kinship-based approach was also extended to be used with time-to-event data: the presence of familial clustering is assessed based on disease incidence rates, thus accounting for the time to disease onset (Kerber, 1995).

Driven by the lack of open access tools for familial aggregation analyses in large pedigrees, we have developed an R package providing this functionality. Besides basic pedigree analysis, sub-setting and plotting methods, it implements the previously published methods based on average kinship and disease incidence rates as well as two novel approaches to detect familial aggregation employing statistics based on kinship coefficients combined with Monte Carlo simulation techniques.

## 2 Implementation

The FamAgg package implements five family aggregation detection methods that can be run on a single family or sets of families and allow stratification according to different conditions such as gender, age and generation.

The *kinship sum* (KS) test assesses whether an affected subject is more closely related to other affected rather than unaffected cases in the pedigree. Let *A* be the set of affected subjects and *N* the number of simulation steps. The kinship sum of subject *i* to all other affected cases is $S_{i}=\sum_{j\in A,\;j\neq i}\Phi_{ij}$, whose null distribution *S* is obtained by *N*-time random sampling of #(*A*) affected cases from the complete pedigree without replacement. An empirical *p*-value for *S_i_* is obtained as *p_i_* = P(*S* ≥ *S_i_*).

In the *kinship group* (KG) test, for each affected individual *i*, its most distant affected relative *k* is identified. We then define a group *G_i_* that includes all individuals *j* such that Φ*_ij_* ≥ Φ*_ik_*. For each group *G_i_*, we calculate two null distributions, based on repeatedly random sampling of #(*A*) affected individuals from the complete pedigree. First, for each group *G_i_* we compute the distribution of the number of affected cases from the random sampling, which allows computing an empirical *p*-value *p_i_* for finding by chance at least the number of observed cases in group *G_i_*. Second, we provide a means to detect clusters of closely related affected family members: for each group *G_i_* we derive the distribution of kinship coefficients Φ*_ia_* from the random sampling for all affected individuals *a*. From this distribution we calculate the empirical *p*-value to find a closer affected relative than in the observed case.

The *genealogical index of familiality* (GIF) test (Hill, 1980) is a pure family-based test. It computes the mean kinship *K_F_* (Malécot, 1948) for a selected family *F*, defined as the average kinship coefficient between all possible pairs of affected individuals *i* and *j*, and creates a null distribution *K* of mean kinships of *N* sets of randomly selected (optionally matched) controls. An empirical *p*-value is derived as *p_F_* = P(*K* ≥ *K_F_*).

The *familial incidence rate* (FIR) approach introduced by Kerber (1995) concentrates on familial aggregation for individuals in longitudinal studies. It is based on the incidence rate *I* *=* *C/T*, where *C* is the number of incident cases and *T* is the total number of years an individual was exposed to the risk of disease (person-years). This measure has been refined by weighting the individual’s contribution and time spent in the study by the kinship coefficient Φ*_ij_* to arrive at a measure of familial incidence rate *FR_i_* for any individual *i*.

Finally, we provide a convenience interface to compute the exact *probability of familial clustering* (PFC) of phenotypes as provided in the gap R package (Yu *et al.*, 2002). It contrasts the number of affected cases against family sizes in a contingency table but the estimation of an exact *p*-value is possible only for families of limited size, due to the high computational demand. The method is based on the exact test for multinomial distributions, and therefore its application to large pedigrees is possible only with the aid of pedigree splitting software such as for example Jenti (Falchi *et al.*, 2008).

With the exception of the GIF method, which identifies aggregation of a trait in the full pedigree all kinship-based methods are applied at the level of individuals and thus allow to identify either individuals in families with significant aggregation (KS test), or groups of highly clustered affected individuals within families (KG test), or assess the risk for individuals given their relation to affected individuals in the pedigree (FIR).

In addition to these familial aggregation methods, FamAgg provides functions to sub-set pedigrees, to identify common ancestors for any given list of individuals, to identify matched controls within pedigrees and to convert pedigrees into graphs, which opens the whole world of graph-theory methods to pedigree analyses. It uses the kinship2 R package (Sinnwell *et al.*, 2014) for kinship coefficient calculation and plotting, and provides a transparent interface to Haplopainter software (Thiele *et al.*, 2005). The open, object-oriented software architecture of the FamAgg package invites contribution of additional tests from the research community. Extensive documentation and examples are distributed with the FamAgg package, which is available as supplementary material.

## 3 Applications

**Fig. 1.**
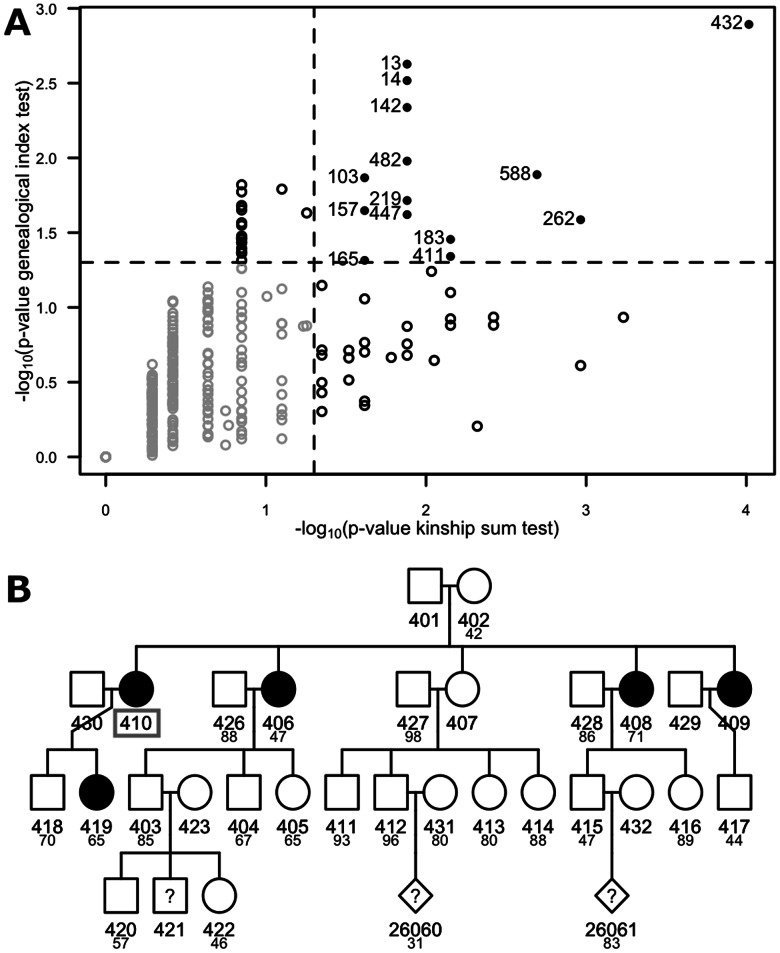
Familial aggregation in the Minnesota Breast Cancer dataset. (**A**) Scatter plot of –log_10_(*p*-values) from the KS test (*x*-axis) and the GIF test (*y*-axis) computed for all 426 families. Given the KS test provides a *p*-value for each affected subject, the lowest *p*-value in each family is displayed. At a significance level of 0.05 (dashed lines), the GIF test identifies 34 families whereas the KS test identifies 42 families. Filled circles and family identifiers are provided for the 14 families when tests are jointly significant. For example, family 432 is top-ranked by both tests: *p*-value = 1.3 × 10^−3^ and 9.6 × 10^−5^ with the GIF and KS test, respectively. Non-significant family clusters are gray shaded. (**B**) Pedigree of family 13, which is ranked second by the GIF test (*p*-value = 2.4 × 10^−3^). The family comprises 29 phenotyped members and includes five affected females. If known, age of cancer onset (cases) or age of demise is indicated below individuals’ identifiers. For subject 410, *S_i_* = 1.0 (0.25 × 3 affected sisters + 0.25 × 1 affected daughter), with *p*-value = 1.3 × 10^−2^. Sisters 406, 408 and 409 have equal *S_i_* = 3 × 0.25 + 0.125 = 0.875 (*p*-value = 2.4 × 10^−2^), as they are aunts of subject 419. The familial incidence rate of individual 410 is *FR_i_* = 8.7 × 10^−3^, which is in the top percentile of all computed values in the Minnesota Breast Cancer dataset

We applied the KS, GIF, KG and FIR tests from the FamAgg package to the publicly available Minnesota Breast Cancer dataset (Sellers *et al.*, 1999), which contains genealogical information from 426 unrelated affected founders whose families entered a longitudinal study on cancer in the state of Minnesota (USA) in 1944. There are 1376 cases spread over these 426 families with a median family size of 53 members, the largest family comprises of 382 individuals in six generations. The performed tests did not utilize sampling stratification and the null distributions were calculated with *N* = 50 000 sampling steps. Runtimes on a single 2.4 GHz processor of a MacBook Pro with 16 GB of memory are as follows: FIR test, 2 s; GIF test, 7 min; KS test, 23 min; and KG test, 3 h. At a significance level of 0.05, the KS test and the GIF test identified 42 and 34 families with a significant enrichment of cases, respectively. Figure 1A highlights the 14 families with filled symbols where both the KS and the GIF tests identified significant familial aggregation. Figure 1B provides an example of a smaller family with breast cancer aggregation. The *p*-values are 2.4 × 10^−3^ for the GIF test and 1.3 × 10^−2^ for individual 410 according to the KS test.

Figure 1 demonstrates that there is a certain agreement between the methods. However, differences in the results from distinct methods are expected as each method is based on a slightly different approach to identify familial aggregation and therefore reports different families at a specified significance level. We recommend bearing in mind the underlying statistical test when interpreting the results of a specific method.

## Supplementary Material

Supplementary Data
