# Supplementary material for: FamAgg: an R package to evaluate familial aggregation of traits in large pedigrees
Source: Bioinformatics. 2016 Jan 22;32(10):1583–5. doi: 10.1093/bioinformatics/btw019 (PMC4866523; doi:10.1093/bioinformatics/btw019)
Supplement: Supplementary Data [file supp_32_10_1583__index.html]

FamAgg: an R package to evaluate familial aggregation of traits in large pedigrees — FamAgg: an R package to evaluate familial aggregation of traits in large pedigrees — Supplementary Data 

# FamAgg: an R package to evaluate familial aggregation of traits in large pedigrees

## Supplementary Data

files

- Supplementary Data - pdf file
