# Supplementary material for: FamAgg: an R package to evaluate familial aggregation of traits in large pedigrees
Source: Bioinformatics. 2016 Jan 22;32(10):1583–5. doi: 10.1093/bioinformatics/btw019 (PMC4866523; doi:10.1093/bioinformatics/btw019)
Supplement: Supplementary Data [file supp_btw019_FamAgg_0_99_9.pdf]

Pedigree Analysis and Familial Aggregation

Contents

1 Introduction **1**

2 Basic pedigree operations **1**
- 2.1 Pedigree analysis methods . . . 7
- 2.2 Additional plotting options . . . 9
- 2.3 Graph utilities . . . 10

3 Importing and exporting pedigree data **12**

4 Testing for familial aggregation **13**
- 4.1 *Genealogical index of familiality* . . . 14
- 4.2 *Familial incidence rate* (FIR) . . . 17
- 4.3 *Kinship sum test* . . . 20
- 4.4 *Kinship group test* . . . 22
- 4.5 Exact inference for family disease clusters (*probability test*) . . . 24

References **25**

Package: *FamAgg* **Authors**: J. Rainer, D. Taliun, C.X. Weichenberger **Modified**: 22 December, 2015 **Compiled**: Tue Dec 22 14:07:39 2015

1 Introduction

This package provides basic pedigree analysis and plotting utilities as well as a variety of methods to evaluate familial clustering of cases from a given trait. Identification of families or groups of individuals within families with significant aggregation of cases can aid also in the selection of interesting and promising individuals for whole genome or exome sequencing projects.

For kinship coefficient calculations and pedigree plotting the package relies and extends the functionality of the `kinship2` package [1].

2 Basic pedigree operations

In the examples below we perform some simple pedigree operations, such as plotting the pedigree for an individual or family, finding the closest common ancestor for a set of individuals in a pedigree or retrieving the identifiers (IDs) of all ancestors for an individual. Basic pedigree information is stored in `FAData` objects, thus we first generate such an object from a subset of the Minnesota Breast Cancer Study provided by the `kinship2` package. In the example below, we generate the `FAData` providing a `data.frame` with the pedigree data, alternatively, the pedigree information could be imported from a file (see Section 3). Upon data set creation the kinship matrix (i.e. a matrix containing the kinship coefficient between each pair of individuals in the whole pedigree) is internally calculated using the functionality from the `kinship2` package [1].

```
library(FamAgg)

data(minnbreast)
## Subsetting to only few families of the whole data set.
```

```
mbsub <- minnbreast[minnbreast$famid %in% 4:14, ]
mbped <- mbsub[, c("famid", "id", "fatherid", "motherid", "sex")]
## Renaming column names.
colnames(mbped) <- c("family", "id", "father", "mother", "sex")
## Defining the optional argument age.
endage <- mbsub$endage
names(endage) <- mbsub$id
## Create the object.
fad <- FAData(pedigree=mbped, age=endage)
```

We can access all the pedigree information stored in this object using the `pedigree` method, but also using `$`. The row names of the pedigree `data.frame` as well as the names of the vectors returned by `$` are the IDs of the individuals in the pedigree.

```
## Use the pedigree method to access the full pedigree
## data.frame,
head(pedigree(fad))
##   family id father mother sex
## 1      4  1     NA     NA   M
## 2      4  2     NA     NA   F
## 3      4  3     25      4   F
## 4      4  4      1      2   F
## 5      4  5      1      2   M
## 6      4  6      1      2   M
```

```
## or access individual columns using $.
## The ID of the father (0 representing "founders"):
head(fad$father)
##  1  2  3  4  5  6
## NA NA 25  1  1  1
```

```
## Mother:
head(fad$mother)
##  1  2  3  4  5  6
## NA NA  4  2  2  2
```

```
## Sex:
head(fad$sex)
## 1 2 3 4 5 6
## M F F F M M
## Levels: M F
```

```
## We can also access the age of each individual, if
## provided.
head(age(fad))
##        1        2        3        4        5        6
##       NA 78.05886 55.50000 48.00000 75.00342 53.63997
```

To extract the pedigree for a single family we can use the `family` method, specifying either the ID of the family or the ID of an individual in the family.

```
## Extract the pedigree information from family "4"...
nrow(family(fad, family=4))
## [1] 43
```

```
head(family(fad, family=4))
##   family id father mother sex
## 1      4  1     NA     NA   M
## 2      4  2     NA     NA   F
## 3      4  3     25      4   F
## 4      4  4      1      2   F
## 5      4  5      1      2   M
## 6      4  6      1      2   M
```

```
## ...which is the same as extracting the family pedigree
## for an individual of this family.
head(family(fad, id=3))
##   family id father mother sex
## 1      4  1     NA     NA   M
## 2      4  2     NA     NA   F
## 3      4  3     25      4   F
## 4      4  4      1      2   F
## 5      4  5      1      2   M
## 6      4  6      1      2   M
```

```
## Note that IDs are internally always converted to character,
## thus, using id=3 and id="3" return the same information.
head(family(fad, id="3"))
##   family id father mother sex
## 1      4  1     NA     NA   M
## 2      4  2     NA     NA   F
## 3      4  3     25      4   F
## 4      4  4      1      2   F
## 5      4  5      1      2   M
## 6      4  6      1      2   M
```

Alternatively, we could subset the `FAData` to individuals of a single family.

```
## Subset the object to a single family.
fam4 <- fad[fad$family == "4", ]
table(fam4$family)
##
##  4
## 43
```

To explore this family we can plot its pedigree. By default, the plotting capabilities of the `kinship2` package are used to plot pedigrees, but alternatively, if all required dependencies are available, the `HaploPainter` [2] perl script (http://haplopainter.sourceforge.net/) can be used instead. The `switchPlotfun` function can be used to switch the plotting back-end. Available arguments are `ks2paint` and `haplopaint` for `kinship2` and `HaploPainter` plotting, respectively. Note however, that `HaploPainter` only allows to export plots to a file, while `kinship2` plotting allows, in addition to export the plot, also to show it as a *standard* `R` plot.

Below we use the `switchPlotfun` to ensure the use of `kinship2` plotting (usually not required) and plot the full available pedigree of individual 3. If the age of individuals is available, it will be plotted below the individual's ID.

```
switchPlotfun("ks2paint")
## By supplying device="plot", we specify that we wish to visualize the
## pedigree in an R plot. This is the default for "ks2paint", anyway.
plotPed(fad, id=3, device="plot")
```

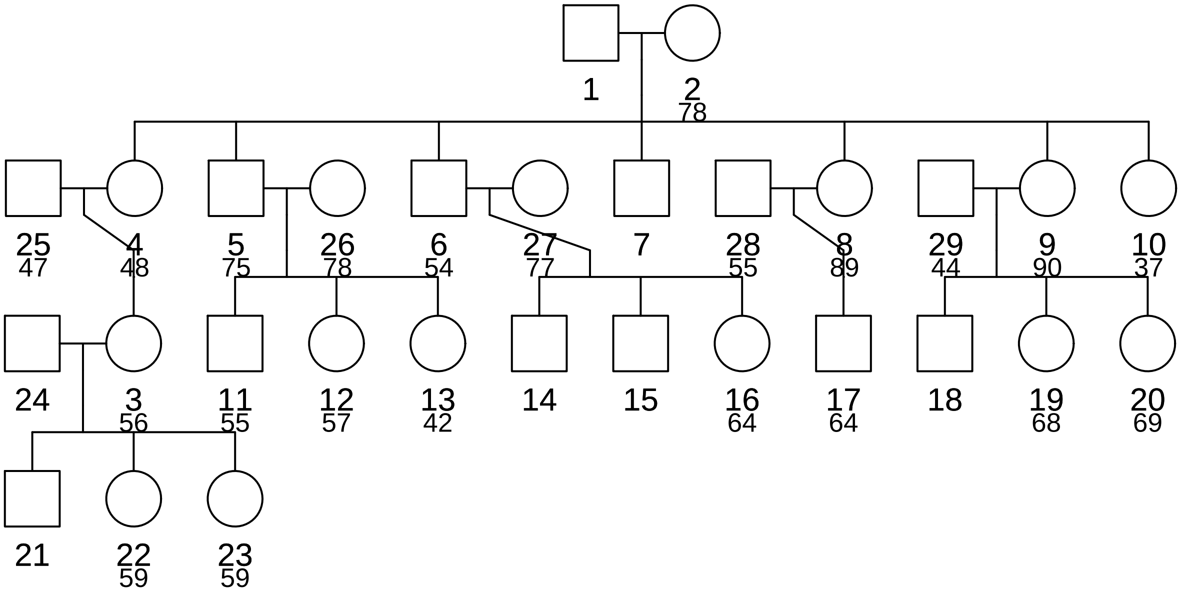

The pedigree for an individual or a list of individuals can be extracted using the `buildPed` method. By default the method first tries to identify all parents up to 3 generations in the pedigree, and subsequently all children of the individuals and all identified parents.

```
## Build the pedigree for individual 3.
fullPed <- buildPed(fad, id="3")
## Removing singletons... none present.
```

```
nrow(fullPed)
## [1] 29
```

Alternatively, we can extract the smallest possible pedigree for a list of individuals by specifying `prune=TRUE`. Internally, the function transforms the pedigree into a graph, tries to find all paths between the individuals and returns the sub-graph of all individuals along with individuals along the paths between them.

```
## Find the subpedigree for individuals 21, 22 and 17.
buildPed(fad, id=c(21, 22, 17), prune=TRUE)
## Removing singletons... none present.
##    family id father mother sex
## 3       4  3     25      4   F
## 4       4  4      1      2   F
## 1       4  1     NA     NA   M
## 8       4  8      1      2   F
## 17      4 17     28      8   M
## 21      4 21     24      3   M
## 22      4 22     24      3   F
## 2       4  2     NA     NA   F
## 25      4 25     NA     NA   M
## 28      4 28     NA     NA   M
## 24      4 24     NA     NA   M
```

And the pedigree plot for that subset of the whole family:

```
plotPed(fad, id=c(21, 22, 17), prune=TRUE)
```

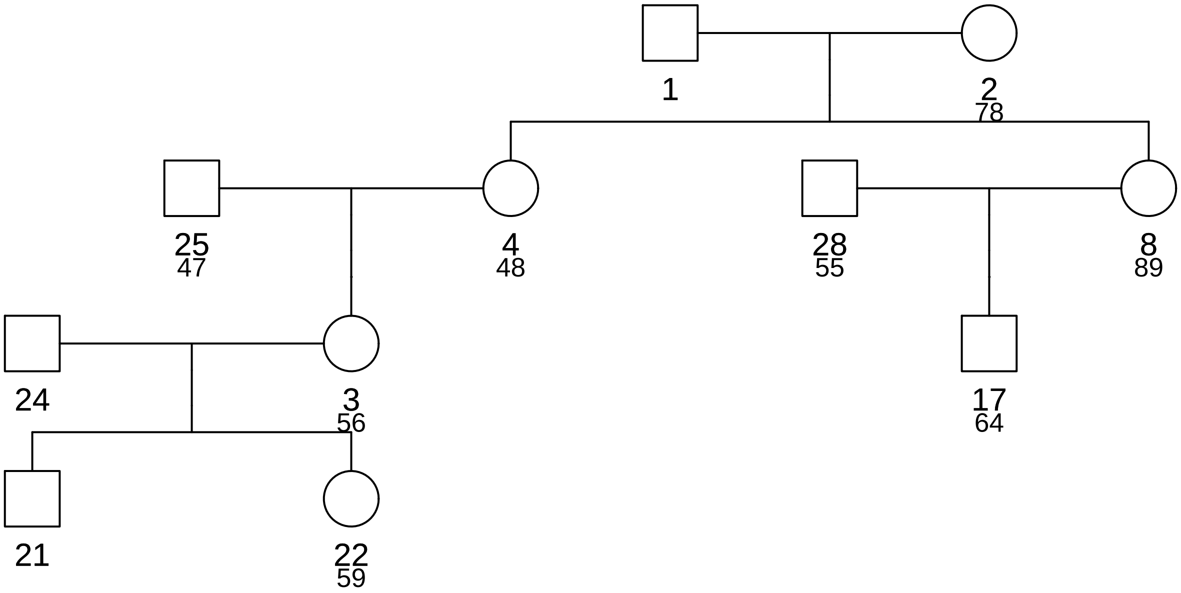

Note that the pedigree returned by the `buildPed` method for an individual might be different than the pedigree of a whole family. The pedigree returned by `buildPed` contains only individuals that share kinship with the specified individual. To exemplify this, we plot the pedigree for the family 14 in the Minnesota Breast Cancer data set. Note that the individuals in the pedigree plot depicted as diamonds are individuals with unknown gender. (The message "Did not plot…" is issued by the `kinship2` plotting function and indicates singletons that are assigned to the family but do neither have parents nor children.)

```
plotPed(fad, family="14", cex=0.4)

## Warning in kinship2::pedigree(id = individual, dadid = father, momid =
## mother, : More than 25% of the gender values are 'unknown'
```

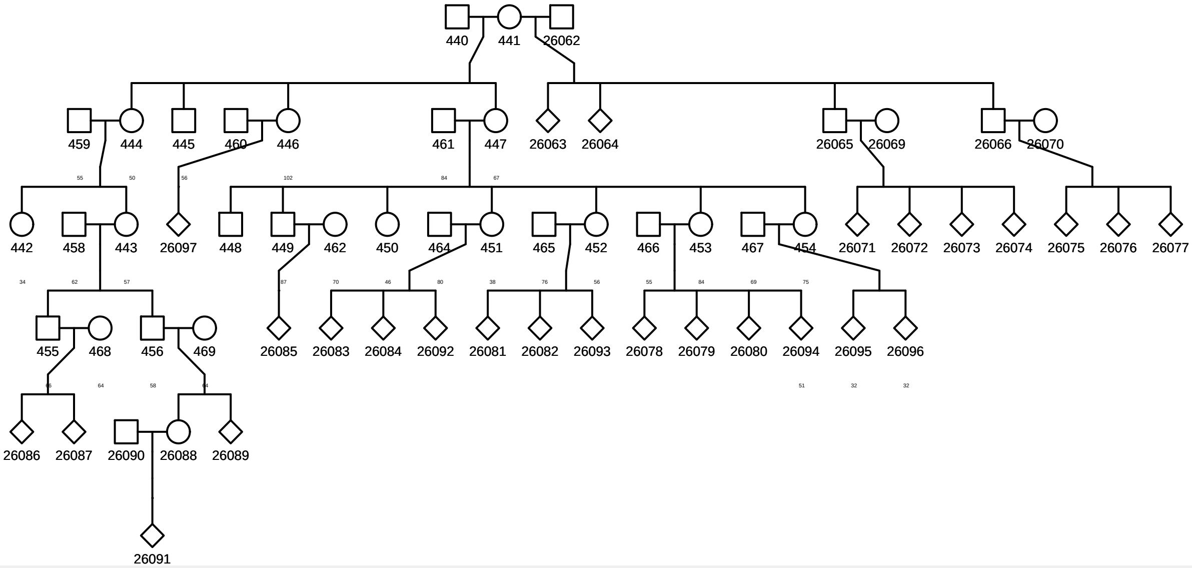


```
## Did not plot the following people: 457 463 470 471 26067 26068 26098 26099
```

In this family, founder 441 is the founder of two family branches. Building the pedigree for individual 440 will not include any of the individuals of the second branch, as he does not share kinship with any of them. The pedigree built for 447 on the other hand contains also individuals from the second branch as she shares kinship with them (*via* her mother 441).

```
## Check if we have individual 26064 from the second branch in the pedigree
## of individual 440.
any(buildPed(fad, id="440")$id == "26064")
## Removing singletons... none present.
## [1] FALSE
```

```
## What for the pedigree of 447?
any(buildPed(fad, id="447")$id == "26064")
## Removing singletons... none present.
## [1] TRUE
```

A family pedigree may consist of many founder couples (i.e. individuals for which neither father nor mother is defined in the pedigree). To identify the pedigree's founder couple (being the couple with the largest number of offspring generations in the pedigree) the `findFounders` method can be used. Note that the function returns always only one couple, even if there might be two founder couples in the family pedigree with the same number of offspring generations.

```
## Find founders for family 4.
findFounders(fad, "4")
## [1] "1" "2"
```

Alternatively, it might be of interest to determine the closest common ancestor between individuals in a pedigree. Below we use the `getCommonAncestor` method to identify the common ancestor for individuals 21, 22 and 17 (which we know from the pedigree a bit above are 1 and 2).

```
## Find the closest common ancestor.
getCommonAncestor(fad, id=c(21, 22, 17))
## [1] "1" "2"
```

Other useful methods are `getChildren`, `getAncestors` and `getSiblings`, that return the children (or all offspring generations up to a specified level), the parents (or all ancestors) or the siblings for the specified individuals, respectively.

```
## Get the children of ID 4.
getChildren(fad, id="4", max.generations=1)
## [1] "3"
```

```
## Get the offsprings.
getChildren(fad, id="4")
## [1] "3"  "21" "22" "23"
```

```
## Get all ancestors.
getAncestors(fad, id="4")
## [1] "1" "2"
```

```
## Get the siblings.
getSiblings(fad, id=c("4"))
## [1] "4"  "5"  "6"  "7"  "8"  "9"  "10"
```

In the whole Minnesota Breast Cancer data set there are 426 families corresponding to 426 founders that had cancer during the screening phase between 1944 and 1952. In the code block below we identify the affected founders per family.

```
## Add the trait information to the FAData object.
cancer <- mbsub$cancer
names(cancer) <- as.character(mbsub$id)
trait(fad) <- cancer
## 521 of in total 521 trait values can be matched to IDs in the pedigree.
```

```
## Identify the affected founders.
## First all affected individuals.
affIds <- affectedIndividuals(fad)
## Identify founders for each family.
founders <- lapply(unique(fad$family), function(z){
    return(findFounders(fad, family=z))
})
names(founders) <- unique(fad$family)

## Track the affected founder.
affFounders <- lapply(founders, function(z){
    return(z[z %in% affIds])
})
## Interestingly, not all founders are affected! It seems in some cases
## parents of the affected participants in the screening phase have also
## been included.
affFounders <- affFounders[unlist(lapply(affFounders, length)) > 0]

## The number of families analyzed.
length(founders)
## [1] 10
```

```
## The number of families with affected founder.
length(affFounders)
## [1] 2
```

Unexpectedly, only in few families one of the founders is affected. For the other families additional (unaffected) ancestors might have been added at a later time point.

Next we get the number of affected individuals that are related to these affected founders.

```
kin2affFounders <- shareKinship(fad, unlist(affFounders))

## How many of these are affected?
sum(kin2affFounders %in% affIds)
## [1] 7
```

```
## How many affected are not related to an affected founder?
sum(!(affIds %in% kin2affFounders))
## [1] 21
```

2.1 Pedigree analysis methods

In this section we perform some more advanced pedigree operations. First, we identify all individuals in the pedigree that share kinship with individual 4.

```
## Get all individuals sharing kinship with individual 4.
shareKinship(fad, id="4")
##  [1] "1"  "2"  "3"  "4"  "5"  "6"  "7"  "8"  "9"  "10" "11" "12" "13" "14"
## [15] "15" "16" "17" "18" "19" "20" "21" "22" "23"
```

Next, we determine generations within the pedigree. Generations can only be estimated for a single family, since in most instances e.g. the year of birth is not available. Thus, generations are estimated considering the relation between individuals, starting from the founder couple, i.e. generation 0, assigning generation 1 to their children and all the mates of

their children and so on. The `estimateGenerations` method calculates such generation numbers for each family defined in the object (or for a single family, if the family ID is provided). The result is returned as a list with the list names corresponding to the family ID and the list elements being the estimated generation numbers (with names corresponding to the ID of the respective individual).

```
## Estimate generation levels for all families.
estimateGenerations(fad)[1:3]
## $`4`
##  1  2  3  4  5  6  7  8  9 10 11 12 13 14 15 16 17 18 19 20 21 22 23 24 25
##  0  0  2  1  1  1  1  1  1  1  2  2  2  2  2  2  2  2  2  2  3  3  3  2  1
## 26 27 28 29 30 31 32 33 34 35 36 37 38 39 40 41 42 43
##  1  1  1  1 NA NA NA NA NA NA NA NA NA NA NA NA NA NA
##
## $`5`
##    44    45    46    47    48    49    50    51    52    53    54    55
##     0     0     2     2     2     2     2     2     2     2     1     3
##    56    57    58    59    60    61    62    63    64    65    66    67
##     3     3     3     3     3     3     3     3     3     2     2     2
##    68    69    70    71    72    73    74    75    76    77    78    79
##    NA     2     1    NA    NA    NA    NA    NA    NA    NA    NA     2
## 26050 26051
##    NA    NA
##
## $`6`
##    80    81    82    83    84    85    86    87    88    89    90    91
##     0     0     2     2     1     1     1     1     1     1     1     1
##    92    93    94    95    96    97    98    99   100   101   102   103
##     1     1     2     2     2     2     2     2     2     3     3     3
##   104   105   106   107   108   109   110   111   112   113   114   115
##     3     2     2     1     1     1    NA    NA    NA    NA     2    NA
##   116   117   118 26052 26053
##    NA    NA    NA     3     3
```

Individuals without generation level (i.e. with an `NA`) are not connected to any other individual in the pedigree (and thus most likely represent errors in the pedigree).

In addition, it is also possible to calculate generation levels relative to a (single) specified individual:

```
gens <- generationsFrom(fad, id="4")
```

We can render these generation numbers into the pedigree:

```
plotPed(fad, family=4, label2=gens)
```

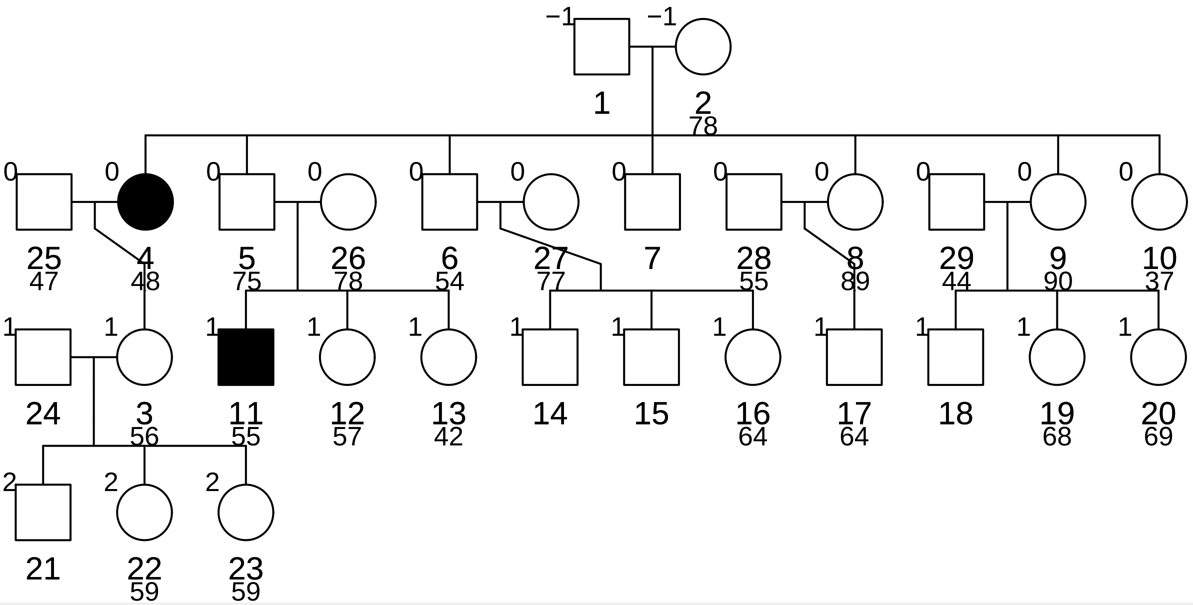


```
## Did not plot the following people: 30 31 32 33 34 35 36 37 38 39 40 41 42 43
```

2.2 Additional plotting options

If a trait information is available it might be of interest to highlight affected individuals in the pedigree. Trait information should always be coded as `0` (or `FALSE`) for unaffected and `1` (or `TRUE`) for affected. In the example below, we use the *cancer* information from the Minnesota Breast Cancer Study.

```
## Extract the cancer trait information.
tcancer <- mbsub$cancer
names(tcancer) <- mbsub$id
## Set the trait.
trait(fad) <- tcancer
```

We can now extract the trait information from the object or identify directly the phenotyped or affected individuals.

```
## Extract the trait information.
head(trait(fad))
## 1 2 3 4 5 6
## 0 0 0 1 0 0
```

```
## We can also extract the IDs of the affected individuals.
head(affectedIndividuals(fad))
## [1] "4"   "11"  "37"  "54"  "84"  "122"
```

```
## Or the IDs of the phenotyped individuals.
head(phenotypedIndividuals(fad))
## [1] "1" "2" "3" "4" "5" "6"
```

Plotting a `FAData` object with trait information results in a pedigree plot with highlighted affected individuals (for `kinship2` pedigree plotting: affected, unaffected and not phenotyped are represented as filled symbols, open symbols and symbols with a question mark inside, respectively).

```
## Plotting the pedigree for family "9".
plotPed(fad, family="9")
```

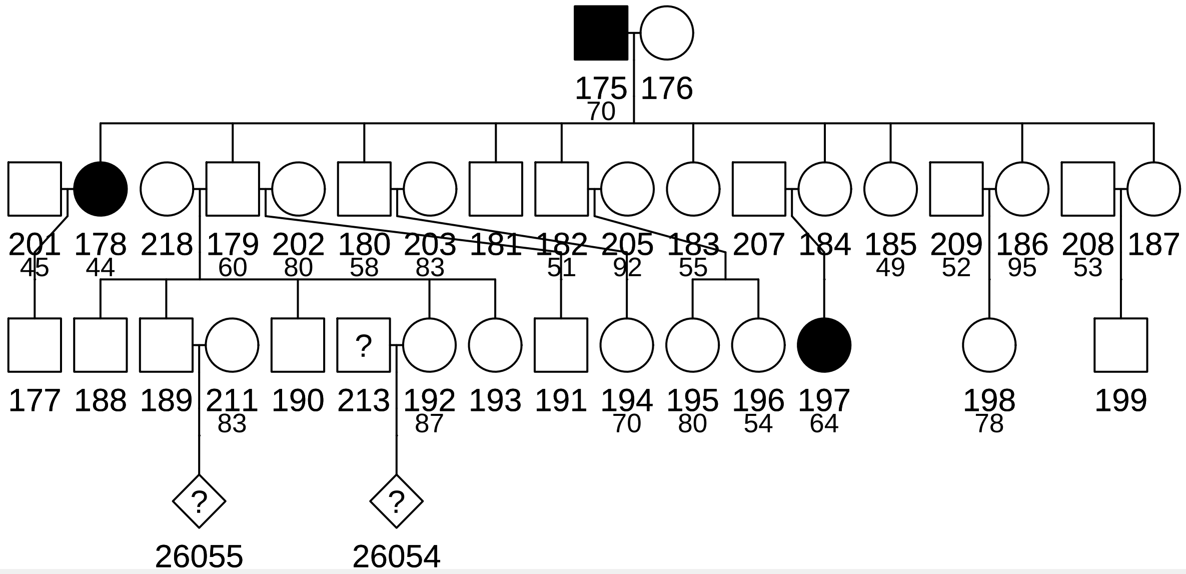


```
## Did not plot the following people: 200 204 206 210 212 214 215 216 217 219
```

In addition, we can manually highlight individuals using the `highlight.ids` argument. For `kinship2` pedigree plotting, a list of length 2 is supported as argument `highlight.ids`, with the first element being plotted on the top left corner of the symbol and the second element on the top right corner.

```
## Plotting the pedigree for family "9".
plotPed(fad, family="9", highlight.ids=list(a=c("185", "201", "198"),
                                  b=c("193")))
```

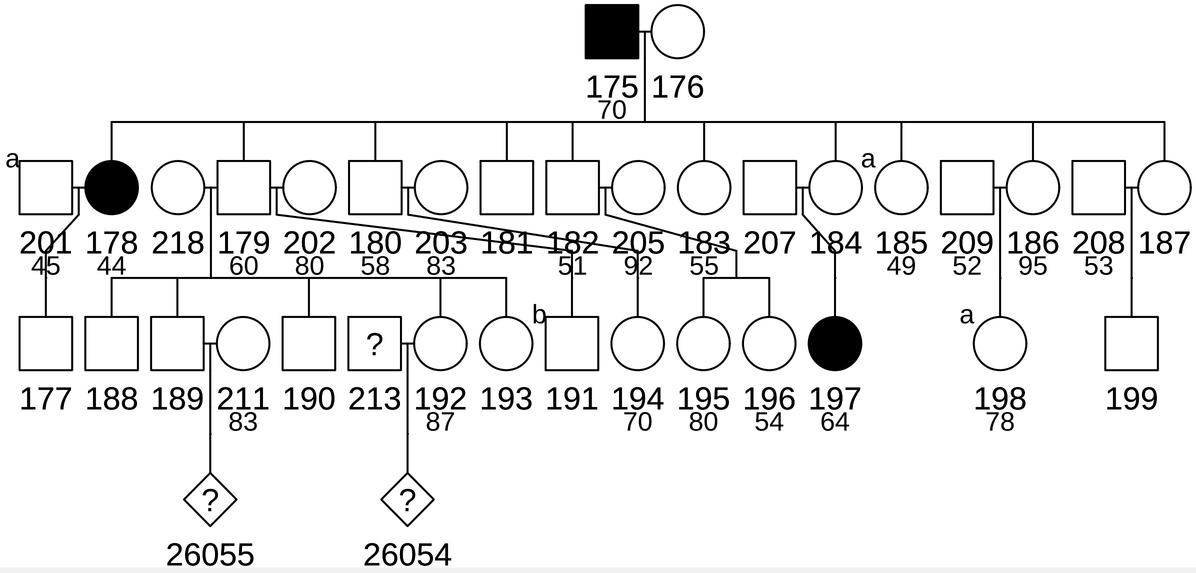


```
## Did not plot the following people: 200 204 206 210 212 214 215 216 217 219
```

An alternative way to highlight individuals or add text to the plot is to use the arguments `label1`, `label2` and `label3` or the `plotPed` method.

2.3 Graph utilities

Pedigrees can also be transformed to graphs using the `ped2graph` function. That way all graph theory methods implemented in e.g. the `igraph` package can be applied to pedigrees.

```
## Transform the full pedigree to a graph.
fullGraph <- ped2graph(pedigree(fad))

## In addition, build the graph for a single family.
singleFam <- ped2graph(family(fad, family=4))
```

We can plot these pedigrees also as graph and could use any of the layout methods provided in the `igraph` package.

```
## Build the layout.
plot(fullGraph)
```

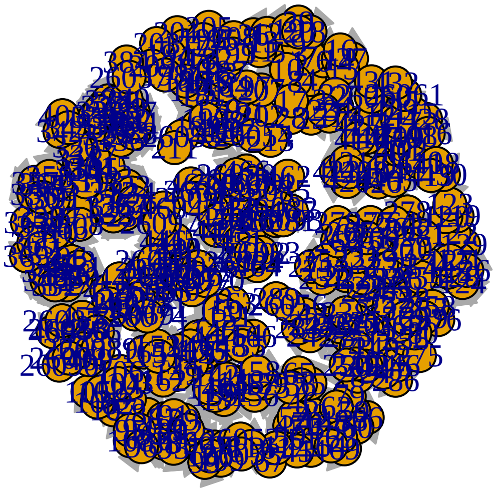

```
lay <- layout_(singleFam, on_grid())
plot(singleFam, layout=lay)
```

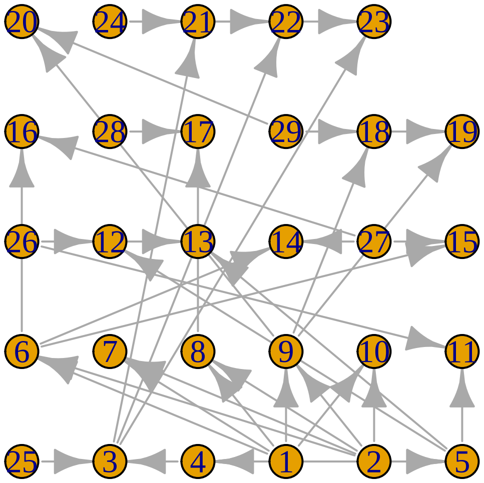

The `connectedSubgraph` function implemented in the `FamAgg` package provides additional functionality to find the smallest connected subgraph of a list of submitted nodes (i.e. individuals).

In the code below we want to extract the smallest possible connected subgraph of the pedigree-graph of family 4 containing individuals 7, 8, 27 and 17.

```
subgr <- connectedSubgraph(singleFam, nodes=c("7", "8", "27", "17"))
```

This is in principle what the `buildPed` method with the option `prune=TRUE` does to find the smallest pedigree for a set of individuals, only that `buildPed` ensures that also eventually missing parents are added.

```
## Plot the graph.
plot(subgr)
```

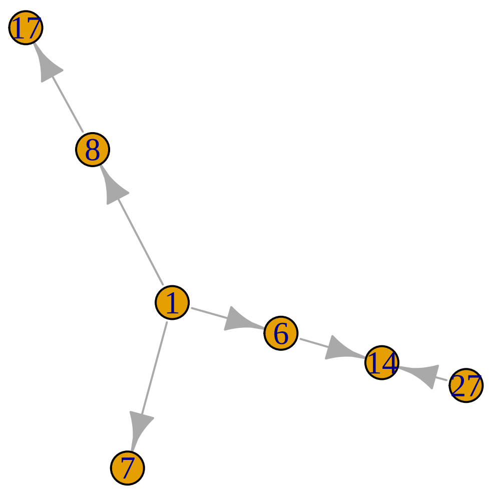


```
## Similar to buildPed/plotPed with prune=TRUE.
plotPed(fad, id=c("7", "8", "17", "27"), prune=TRUE)
## Removing singletons... none present.
```

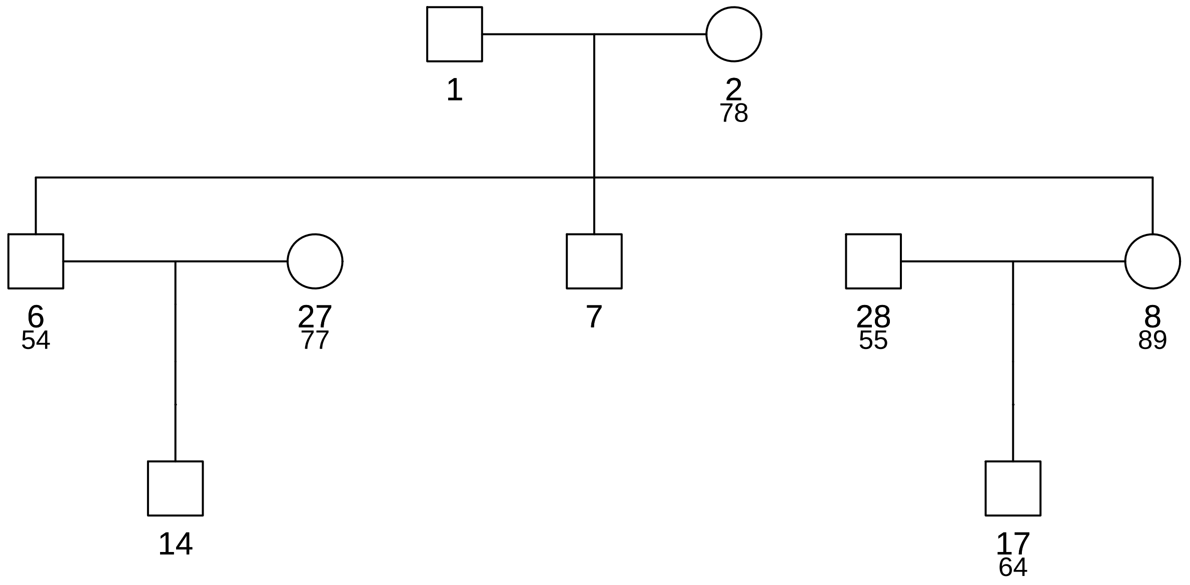

3 Importing and exporting pedigree data

Besides providing the pedigree data as a `data.frame`, the `FAData` constructor can also read pedigree data from various file formats, such as plink [3] *ped* or *fam* files (http://pngu.mgh.harvard.edu/~purcell/plink/data.shtml) or generic text files.

```
## Import a "ped" file.
pedFile <- system.file("txt/minnbreastsub.ped.gz", package="FamAgg")
## Quick glance at the file.
readLines(pedFile, n=1)
```

```
## [1] "4\t1\t0\t0\t1\t1"
```

```
fad <- FAData(pedFile)

head(pedigree(fad))
##   family id father mother sex affected
## 1      4  1   <NA>   <NA>   M        0
## 2      4  2   <NA>   <NA>   F        0
## 3      4  3     25      4   F        0
## 4      4  4      1      2   F        1
## 5      4  5      1      2   M        0
## 6      4  6      1      2   M        0
```

Alternatively, we can import pedigree data from generic input files.

```
## Create the FAData by reading data from a txt file.
pedFile <- system.file("txt/minnbreastsub.txt", package="FamAgg")
fad <- FAData(pedigree=pedFile, header=TRUE, id.col="id",
          family.col="famid", father.col="fatherid",
          mother.col="motherid")
```

And we can export pedigree data again using the `export` method. In the example below, we subset the whole pedigree to the pedigree of family 4 and export this as a *ped* file.

```
tmpF <- tempfile()

## Subset the pedigree to family 4
fam4 <- fad[fad$family == 4, ]

## Export data in ped format.
export(fam4, tmpF, format="ped")
```

4 Testing for familial aggregation

Familial aggregation aims to identify families within large ancestral pedigrees that show a non-random aggregation of traits.

As an example, we analyze here data from the Minnesota Breast Cancer Record, which is provided by the `kinship2` package. In brief, this data set consists of genealogical information from 426 unrelated founders diagnosed with breast cancer whose families entered a longitudinal study on cancer in the state of Minnesota (USA) in 1944. Cancer cases are encoded with a 1 in column `cancer` in the `minnbreast` `data.frame`. Note however that, besides breast cancer, also prostate cancer cases are reported. This unfortunately causes a systematic bias in the data set as families were only included if a founder was diagnosed with breast cancer, but all occurrences of both breast and prostate cancer are reported. Based on this bias many of the results below should be taken with caution. Another important information is provided in column `endage`, which represents either the age of cancer onset, the age at the end of the study or the age at death of the participant.

Note that, to reduce computation time, we perform the analysis only on a subset of families from the Minnesota Breast Cancer record and reduce the number of simulation runs. We specifically selected some families with a high percentage of cancer cases, thus, the analysis presented here is biased. Also, in a real analysis you should increase the `nsim` argument.

```
library(FamAgg)
set.seed(18011977)
data(minnbreast)
## Subset the dataset to reduce processing time.
```

```
mbsub <- minnbreast[minnbreast$famid %in% c(4:60, 432), ]
## Uncomment the line below to use the whole dataset instead.
## mbsub <- minnbreast

## Define the number of simulations we perform.
## nsim <- 10000
nsim <- 1000

mbped <- mbsub[, c("famid", "id", "fatherid", "motherid", "sex")]
## Renaming column names.
colnames(mbped) <- c("family", "id", "father", "mother", "sex")
## Create the FAData object.
fad <- FAData(pedigree=mbped)

## Define the trait.
tcancer <- mbsub$cancer
names(tcancer) <- as.character(mbsub$id)
```

In the following section we analyze the data set first using the *genealogical index* method [4] (Section 4.1), then we estimate the per-individual risk of disease using the *familial incidence rate* (FIR, also abbreviated as *FR* in the original work) [5] (Section 4.2) and apply our *kinship sum test* to identify affected individuals exhibiting a higher relationship to other affected individuals than what would be expected by chance (Section 4.3). Subsequently, we apply our *kinship group test* (Section 4.4) that allows to identify highly clustered affected individuals within families.

In Section 4.5 we apply the *probability test* based on the method from Yu et al [6] for inference on family disease clusters. We use the corresponding implementation in the gap package. However, currently gap cannot be directly applied to large pedigrees due to a specific limitation in the implementation.

The *genealogical index of familiality*, the *familial incidence rate* and the *probability test* are well established methods while the *kinship sum test* and the *kinship group test* are novel approaches presented here for the first time.

4.1 *Genealogical index of familiality*

We next calculate the *genealogical index of familiality* (GIF) [4] (referred to as the *genealogical index* in the original work) for cancer occurrence in a subset of the Minnesota Breast Cancer Record data set. For a given trait (e.g. whether or not an individual was diagnosed with a certain type of cancer), the method computes the mean kinship between affected individuals (cases) in the whole pedigree along with mean kinship values of randomly drawn sets of individuals. The distribution of average kinship values among the control sets is then used to estimate the probability that the observed level of kinship among the cases is due to chance.

Below, we perform the analysis using the `genealogicalIndexTest` method on the `cancer` trait. In its default setting, the `genealogicalIndexTest` function uses all phenotyped individuals in the pedigree as control population from which sets of random samples equal in size to the number of affected are drawn.

Note that by default the function excludes all singletons (i.e. unconnected individuals in the pedigree) from the analysis. Changing the argument `rm.singletons` to `FALSE` will estimate the GIF on the full data set.

```
## Calculate the genealogical index of familiality.
gi <- genealogicalIndexTest(fad, trait=tcancer, traitName="cancer", nsim=nsim)
## Removing singletons... 789 removed.
## Cleaning data set (got in total 2489 individuals):
##  * not phenotyped individuals among selected controls... 715 removed.
## Done
```

```
## Display the result.
result(gi)
```

```
##   trait_name total_phenotyped total_affected entity_id entity_ctrls
## 1     cancer             2202            154         1         1774
##   entity_affected genealogical_index pvalue padj
## 1             132           334.6923      0    0
```

The column *genealogical index* of the result `data.frame` shown above represents the mean kinship between all pairs of affected individuals in the pedigree multiplied by 100000 for easier interpretation. Thus, according to the GIF test, a clustering of cancer cases is present in the analyzed pedigree. The output messages from the method call indicate that some individuals have been excluded from the test since they were either not phenotyped in the trait (i.e. have a missing value in trait), or are not *connected* in the family pedigree (do not share kinship with any other individual in the pedigree after removing non-phenotyped individuals).

The genealogical index of familiality implementation in this package adds some more flexibility to the original approach. The definition of the appropriate set of control individuals from which random samples are drawn can be specified with the `controlSetMethod` argument. Also, it is possible to perform a stratified sampling, e.g. if the group of affected cases in a pedigree consists of 5 female and 3 male individuals, submitting the sex of each individual in the pedigree with the argument `strata` (i.e. `strata=fad$sex`, with `fad` being the `FAData` object on which the analysis is performed) allows the function to define random control sets with the same proportion of male/female individuals.

In the next example, we use the `getSexMatched` function to define the set of control individuals and also the `getExternalMatched` submitting the gender information of each individual. The results from both approaches are essentially identical, and in the present data set not that useful, as the Minnesota Breast Cancer data set lists both, breast cancer and prostate cancer in column `cancer`, thus, the set of control individuals will contain all individuals with known sex.

```
## Calculate the genealogical index of familiality using random sampling from
## a sex matched control set.
giSexMatch <- genealogicalIndexTest(fad, trait=tcancer, traitName="cancer", nsim=nsim,
                controlSetMethod="getSexMatched")

## Use an external vector to perform the matching.
## The results are essentially identical.
giExtMatch <- genealogicalIndexTest(fad, trait=tcancer, traitName="cancer", nsim=nsim,
                controlSetMethod="getExternalMatched", match.using=fad$sex)
```

Note that any matching or stratified sampling can lead to the exclusion of individuals with missing values in either the matching criteria or the strata.

In the Minnesota Breast Cancer data set, the number of prostate cancer cases is much lower than the number of breast cancer cases, thus, simple random sampling might result in an biased genealogical index of familiality estimate since about the same proportion of male and female individuals will be sampled. To account for such cases a stratified sampling, as performed below, can be used instead.

```
## Evaluate the proportion of male and femal cases.
table(gi$sex[affectedIndividuals(gi)])
##
##   M   F
##  19 133
```

```
## We can use the gender information to perform stratified sampling, i.e.
## in each permutation a random set of 3 male and 15 females will be selected.
giStrata <- genealogicalIndexTest(fad, trait=tcancer, traitName="cancer", nsim=nsim,
                  strata=fad$sex)

result(giStrata)
##   trait_name total_phenotyped total_affected entity_id entity_ctrls
## 1     cancer             2202            154         1         1771
```

```
##   entity_affected genealogical_index pvalue padj
## 1             132           334.6923      0    0
```

Finally, we plot the result from the simulation. The blue vertical line in the plot below represents the mean kinship value between all affected individuals in the pedigree. The distribution of mean kinship values from the 1000 randomly drawn sets are shown in grey color.

```
## Plot the result.
plotRes(giStrata)
```

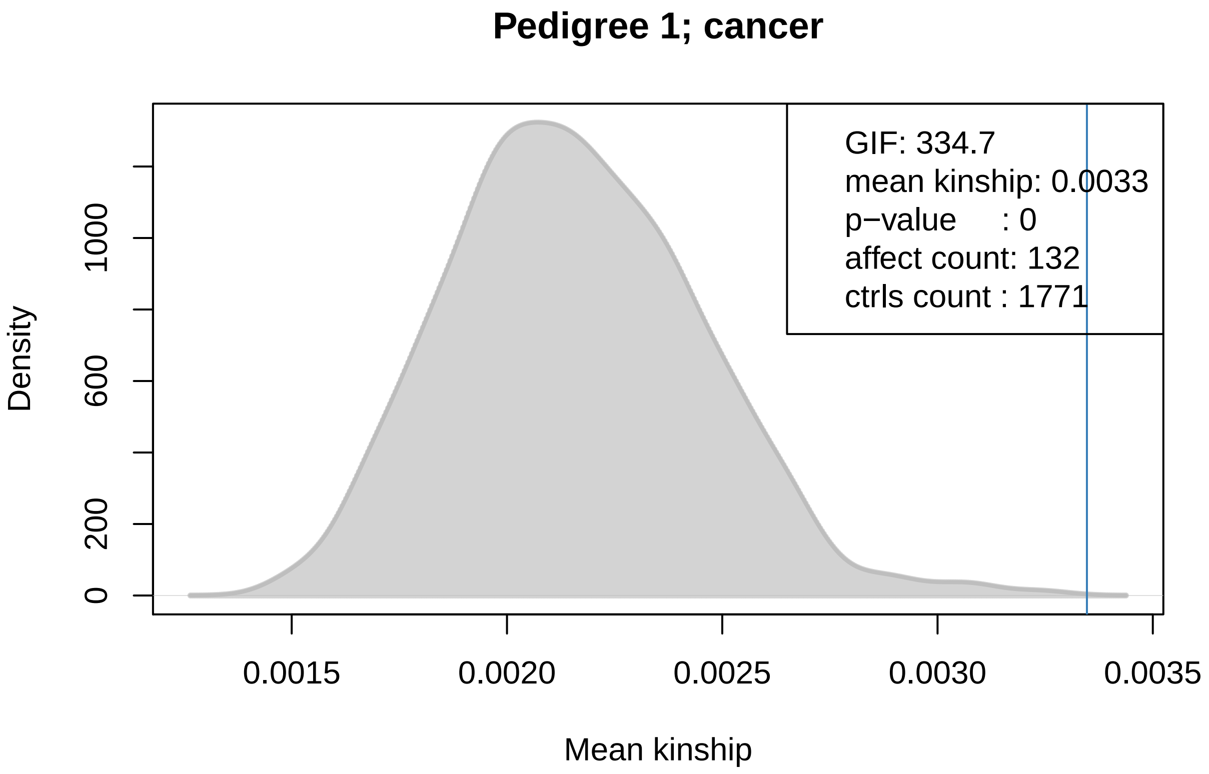

The genealogical index of familiality can also be estimated by the `gif` function from the gap R-package. Below we calculate the estimate using both methods and compare the resulting estimate. Note that the `gif` method reports only the genealogical index of familiality estimate but does not estimate significance.

```
library(gap)

## Adding the trait information, so the extracted pedigree data.frame will
## also contain a column "affected" with that information.
trait(fad) <- tcancer

## Extract the pedigree and re-format it for the gif function.
pedi <- pedigree(fad)
## Remove singletons.
pedi <- removeSingletons(pedi)
pedi[is.na(pedi$father), "father"] <- 0
pedi[is.na(pedi$mother), "mother"] <- 0

## Identify the affected individuals.
affIds <- as.numeric(pedi$id[which(pedi$affected == 1)])

## Execute the gif method contained in the gap package.
```

```
gifRes <- gif(pedi[, c("id", "father", "mother")], affIds)

## Calculate the GIF using FamAgg's genealogicalIndexTest.
gifT <- genealogicalIndexTest(fad, trait=tcancer, nsim=100)

## Comparing the results:
gifRes[[1]] == result(gifT)$genealogical_index
## [1] TRUE
```

Thus, the GIF estimate from the gap package is identical to the one from the FamAgg package.

In the examples above, we tested for an enrichment of cancer cases in the full data set, i.e. across all families. In addition, we can perform the test individually for each family, by setting the perFamilyTest parameter of the genealogicalIndexTest to TRUE, and thus test for a clustering of cancer cases within each family.

```
## Perform the analysis (no strata etc) separately for each family.
giFam <- genealogicalIndexTest(fad, trait=tcancer, nsim=nsim,
                   perFamilyTest=TRUE, traitName="Cancer")

## Display the result from the analysis.
head(result(giFam))
##     trait_name total_phenotyped total_affected entity_id entity_ctrls
## 13      Cancer             2202            154        13           29
## 14      Cancer             2202            154        14           31
## 432     Cancer             2202            154       432          106
## 40      Cancer             2202            154        40           39
## 30      Cancer             2202            154        30           25
## 48      Cancer             2202            154        48           58
##     entity_affected genealogical_index pvalue  padj
## 13                5          21250.000  0.000 0.000
## 14                5          21250.000  0.001 0.015
## 432              15           9940.476  0.003 0.030
## 40                3          20833.333  0.032 0.240
## 30                3          25000.000  0.041 0.246
## 48                3          16666.667  0.083 0.415
```

4.2 *Familial incidence rate* (FIR)

A per-individual risk of e.g. disease can be calculated using the *familial incidence rate* (FIR, abbreviated as *FR* in the original work) [5]. This measure considers the kinship of each individual with any affected in a given trait in the pedigree and the time at risk for each individual. Thus, the FIR is an estimate for the risk per gene-time for each individual given the disease-experience in the cohort.

As *time at risk* for each individual we use the endage column in the Minnesota Breast Cancer data set, which represents the participant's age at the last follow-up or at cancer incidence. This estimate of time at risk is rather crude and in a real life situation a better, more accurate, estimate that is based e.g. on the birth dates and dates of last follow up or incidence might be used instead. See the help of functions estimateTimeAtRisk and sliceAge for details and options related to *time at risk*.

```
## Estimate the risk for each individual using the familial incidence rate method.
## We use the endage provided in the Minnesota Breast Cancer Record as
## a measure for time at risk.
fr <- familialIncidenceRate(fad, trait=tcancer, timeAtRisk=mbsub$endage)
```

```
## Cleaning data set (got in total 3278 individuals):
##  * not phenotyped individuals... 1076 removed.
##  * individuals with unknown time at risk... 622 removed.
##  * singletons (also caused by previous subsetting)... 466 removed.
## Done
```

A note on singletons: for all per-individual measures unconnected individuals within the pedigree are automatically excluded from the calculations as no kinship-based statistics can be estimated for them (they do, by definition, not share kinship with any other individual in the pedigree, thus their kinship coefficient with any other individual in the pedigree will be 0). Note also that the removal of e.g. not phenotyped individuals prior to the calculation can also *generate* singletons, that additionally become removed. This removal results in an estimate with the value `NA` for all singletons as well as not phenotyped individuals.

Next, we calculate the mean FIR within each family and plot this information.

```
## Split the FIR by family and average the values within each.
frFam <- split(fr, f=fad$family)
frFamAvg <- lapply(frFam, mean, na.rm=TRUE)

## Sort and plot the averages.
frFamAvg <- sort(unlist(frFamAvg), decreasing=TRUE)
plot(frFamAvg, type="h", xaxt="n", xlab="", ylab="mean FIR",
     main="Per family averaged familial incidence rate")
axis(side=1, las=2, at=1:length(frFamAvg), label=names(frFamAvg))
```

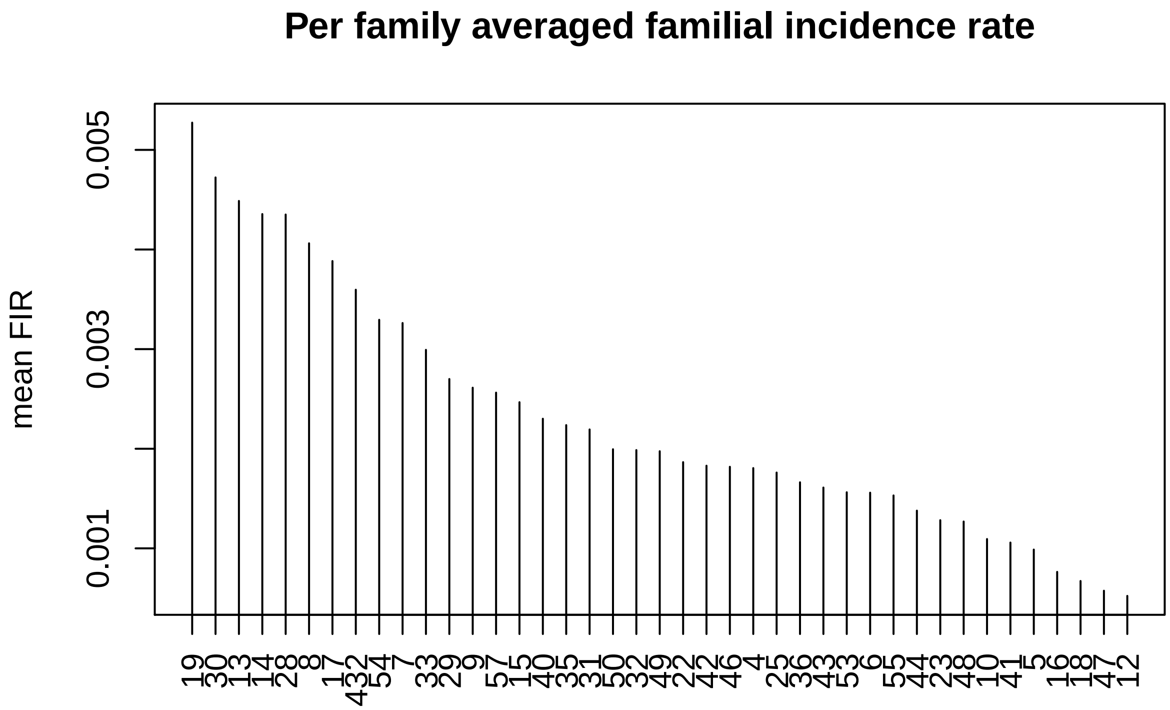

Not unexpectedly, individuals in some families have on average a higher familial incidence rate, and thus a higher risk of cancer than others.

In the next example, we calculate the familial incidence rate assessing in addition the significance of each estimate using Monte Carlo simulations. This extension to the original approach from Kerber [5] does also allow stratified sampling.

```
## Estimate the risk for each individual using the familial incidence rate method.
## We use the endage provided in the Minnesota Breast Cancer Record as
## a measure for time at risk.
```

```
frTest <- familialIncidenceRateTest(fad, trait=tcancer, traitName="cancer",
                    timeAtRisk=mbsub$endage, nsim=nsim)
```

The familial incidence rate can be extracted easily from the result object using the `familialIncidenceRate` method or using `$fir`. Also, the empirical p-value from the simulation analysis and the time at risk can be accessed using the $ operator (i.e. using `$pvalue`, `$tar` or `$timeAtRisk`, respectively).

```
head(familialIncidenceRate(frTest))
##           1           2           3           4           5           6
##          NA 0.002278208 0.002365165 0.000670492 0.002709228 0.002098398
```

```
head(frTest$fir)
##           1           2           3           4           5           6
##          NA 0.002278208 0.002365165 0.000670492 0.002709228 0.002098398
```

Finally, we inspect the results from the analysis.

```
head(result(frTest))
##       trait_name total_phenotyped total_affected total_tested    id family
## 418       cancer             2202            154         1114   418     13
## 17545     cancer             2202            154         1114 17545    432
## 17546     cancer             2202            154         1114 17546    432
## 17550     cancer             2202            154         1114 17550    432
## 17548     cancer             2202            154         1114 17548    432
## 17547     cancer             2202            154         1114 17547    432
##               fir pvalue padj
## 418   0.007164812      0    0
## 17545 0.006722134      0    0
## 17546 0.006709385      0    0
## 17550 0.005765806      0    0
## 17548 0.005746871      0    0
## 17547 0.005647192      0    0
```

We can also identify the families containing individuals with a significant FIR.

```
frRes <- result(frTest)
frSig <- frRes[which(frRes$padj < 0.05), ]

## Split by family.
frFam <- split(frSig, frSig$family)
frRes <- data.frame(family=names(frFam), no_sign_fir=unlist(lapply(frFam, nrow)))
## Determine the number of phenotyped and affected individuals per family.
noPheNAff <- sapply(names(frFam), function(z){
    fam <- family(frTest, family=z)
    return(c(no_pheno=sum(!is.na(fam$affected)),
         no_aff=length(which(fam$affected == 1))
         ))
})
frRes <- cbind(frRes, t(noPheNAff))

## Display the number of phenotyped and affected individuals as well as
## the number of individuals within the families with a significant FIR.
frRes[order(frRes[, "no_sign_fir"], decreasing=TRUE), ]
##     family no_sign_fir no_pheno no_aff
## 432    432          10      123     15
## 13      13           1       34      5
```

Again, we can see that we have an enrichment of affected cases in families 432 and 13.

4.3 *Kinship sum test*

Next, we use the *kinship sum test* that evaluates familial aggregation based on the sum of kinship values between affected cases. The test identifies affected individuals exhibiting a higher relationship to other affected individuals than would be expected by chance. By specifying the `strata` we perform sex-stratified random sampling, i.e. ensure that the proportion of male and female individuals in each randomly sampled group matches the corresponding proportions in the *real*, observed, affected.

```
## Perform the kinship sum test.
kinSum <- kinshipSumTest(fad, trait=tcancer, traitName="cancer",
             nsim=nsim, strata=fad$sex)
## Cleaning data set (got in total 3278 individuals):
##  * not phenotyped individuals...
##  1076 removed
##  * unaffected individuals without valid strata values... 5 removed.
##  * affected individuals without valid strata values... 2 removed.
## Done
```

```
head(result(kinSum))
##       trait_name total_phenotyped total_affected affected_id family
## 17528     cancer             2202            154       17528    432
## 17517     cancer             2202            154       17517    432
## 17529     cancer             2202            154       17529    432
## 17547     cancer             2202            154       17547    432
## 17548     cancer             2202            154       17548    432
## 17549     cancer             2202            154       17549    432
##       affected kinship_sum        freq        pvalue        padj
## 17528      152        2.00 0.003289474 0.00005263158 0.003285714
## 17517      152        1.75 0.001973684 0.00015131579 0.003285714
## 17529      152        1.75 0.001973684 0.00015131579 0.003285714
## 17547      152        1.75 0.001973684 0.00015131579 0.003285714
## 17548      152        1.75 0.001973684 0.00015131579 0.003285714
## 17549      152        1.75 0.001973684 0.00015131579 0.003285714
```

Next, we identify those individuals that have a significant kinship sum accepting a 10% false discovery rate (FDR).

```
## Extract the IDs of the individuals with significant kinship. By default, the raw
## p-values are adjusted for multiple hypothesis testing using the method from
## Benjamini and Hochberg.
kinSumRes <- result(kinSum)
kinSumIds <- as.character(kinSumRes[kinSumRes$padj < 0.1, "affected_id"])

## From which families are these?
table(kinSumRes[kinSumIds, "family"])
##
## 432
##  12
```

Thus, most of the identified significant individuals are from one family. Next, we compare the FIR scores of affected or unaffected (but phenotyped) individuals in this family to the FIR scores of affected or unaffected individuals of all other families.

```
## Get the familial ratio of the significant in this family, of all in this family,
## and of all others.
famId <- kinSumRes[1, "family"]

## Extract the family.
fam <- family(kinSum, family=famId)

## Stratify individuals in affected/unaffected.
strat <- rep("All, unaff.", length(kinSum$id))
strat[which(kinSum$affected > 0)] <- "All, aff."
strat[kinSum$id %in% fam$id] <- paste0("Fam ", famId, ", unaff.")
strat[kinSum$id %in% fam$id[which(fam$affected > 0)]] <- paste0("Fam ",famId,", aff.")

famData <- data.frame(fr=fr, group=strat)
boxplot(fr~group, data=famData, na.rm=TRUE, ylab="FIR",
    col=rep(c("#FBB4AE", "#B3CDE3"), 2))
```

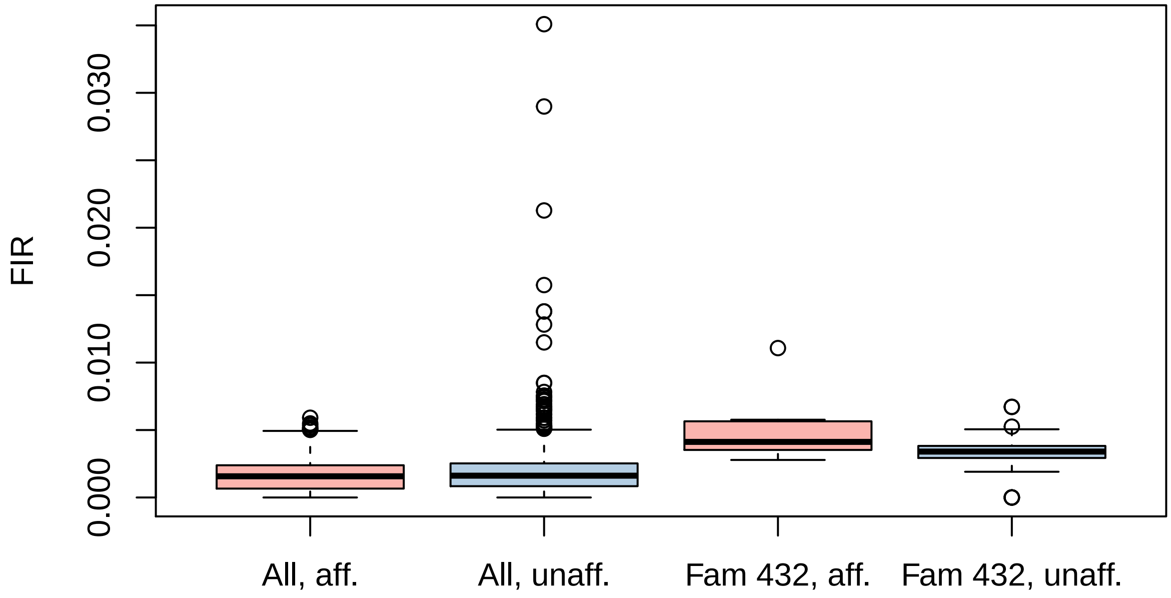

As expected, the familial incidence rate (i.e., in the present data set, the risk of individuals to get cancer, given their kinship to other cancer cases) for individuals (whether affected or yet unaffected) in this family is higher than in the data set analyzed here.

Next, we plot the pedigree of this family.

```
## Plot the pedigree for the family of the selected individual removing
## all individuals that were not phenotypes.
plotPed(kinSum, id=kinSumIds[1], cex=0.3, only.phenotyped=TRUE)
```

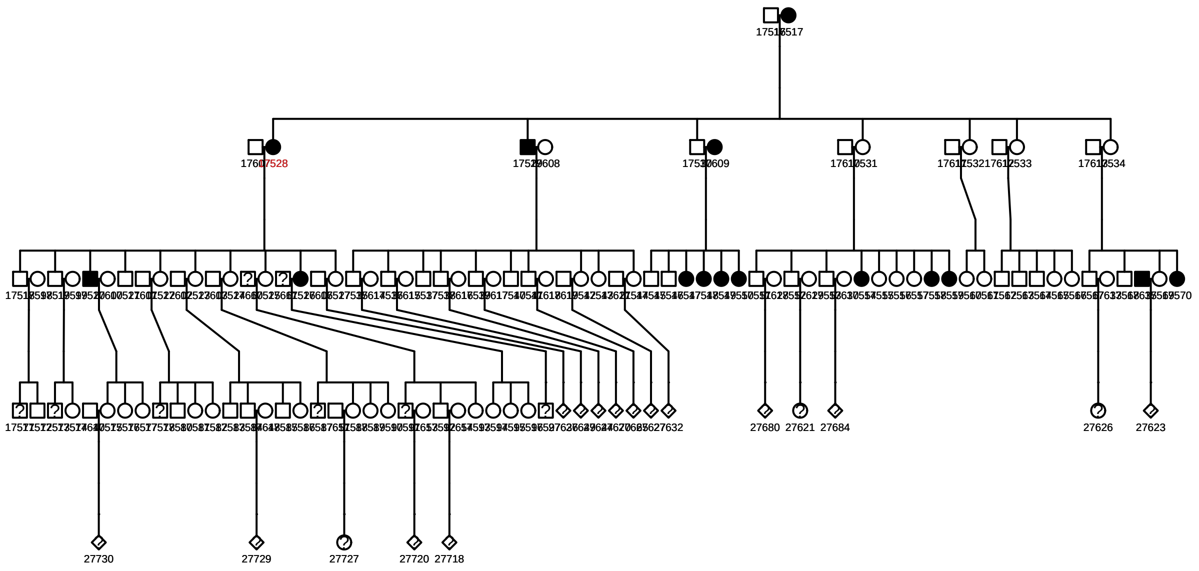

And finally, also plot the kinship sum for the individuals with the largest kinship sum in relation to the *expected* kinship sums from the Monte Carlo simulations.

```
plotRes(kinSum, id=kinSumIds[1])
```

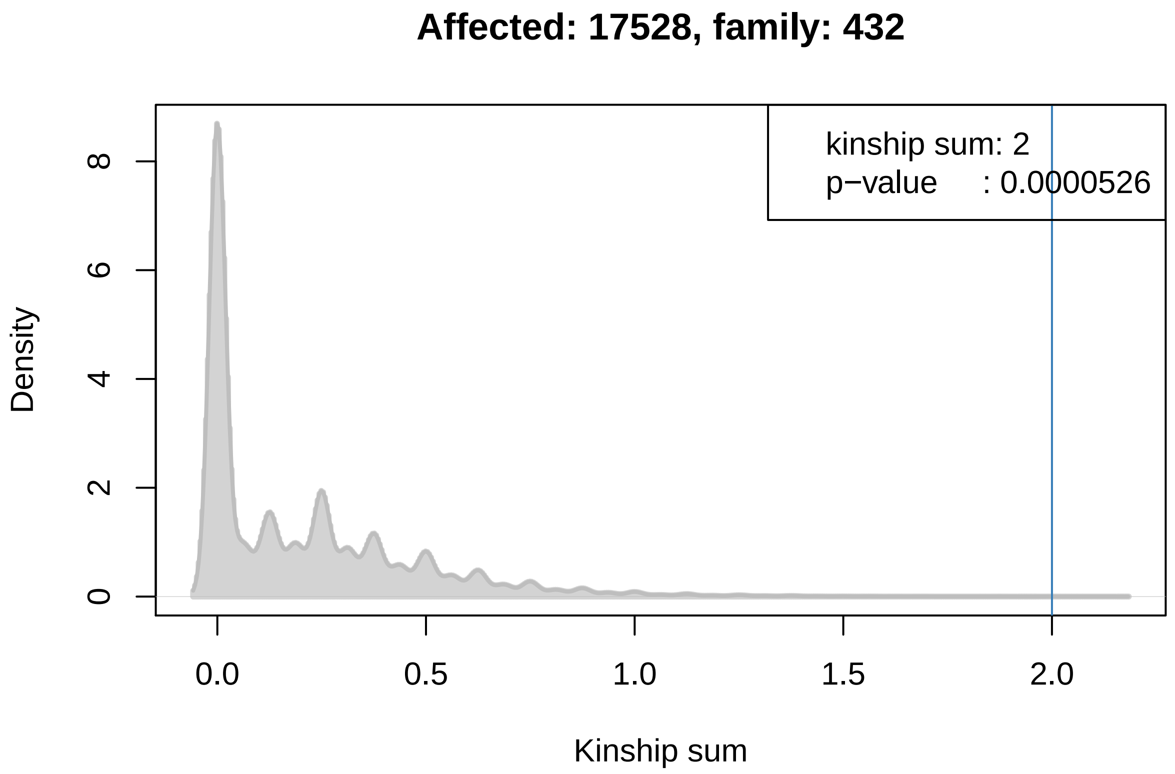

4.4 *Kinship group test*

Here we apply the *kinship group test* to the data set. This test first defines for each affected individual a group of individuals considering only individuals that are as closely related as the most distant affected individual. For each of these kinship groups two tests are then performed, one by comparing the mean kinship among affected in the group with the mean kinship from Monte Carlo simulations (ratio test) and one evaluating the largest observed kinship value between

affected individuals with those of random samples from the simulation (kinship group test).

In the example below we specify again the `strata` argument and thus perform sex-stratified random sampling.

```
## Calculate the kinship test.
kinGroup <- kinshipGroupTest(fad, trait=tcancer, traitName="cancer",
                nsim=nsim, strata=fad$sex)
head(result(kinGroup))
##       trait_name total_phenotyped total_affected phenotyped affected
## 410       cancer             2202            154        650      103
## 447       cancer             2202            154        650      103
## 17609     cancer             2202            154        650      103
## 17517     cancer             2202            154        650      103
## 17547     cancer             2202            154        650      103
## 2223      cancer             2202            154        650      103
##       group_id family group_phenotyped group_affected ratio_pvalue
## 410        410     13                8              5        0.000
## 447        447     14               12              5        0.000
## 17609    17609    432                6              5        0.000
## 17517    17517    432               53             13        0.000
## 17547    17547    432               55             14        0.000
## 2223      2223     53               17              4        0.006
##       ratio_padj mean_kinship kinship_pvalue kinship_padj
## 410        0.000    0.2500000          0.000  0.000000000
## 447        0.000    0.2500000          0.000  0.000000000
## 17609      0.000    0.2500000          0.000  0.000000000
## 17517      0.000    0.1458333          0.000  0.000000000
## 17547      0.000    0.1346154          0.000  0.000000000
## 2223       0.034    0.2500000          0.001  0.005666667
```

The kinship group test finds a significant aggregation of cases in family 432. In fact, as we see further below, the test identified a subgroup in this family with an exceptional high proportion of cases.

Below, we summarize the results further by listing the total number of families in the pedigree and the number of families in which kinship groups with significant kinship p-value and significant ratio p-value (both at a 5% FDR).

```
kinGroupRes <- result(kinGroup)
## Create a data.frame with the summarized results.
resTab <- data.frame(total_families=length(unique(kinGroup$family)),
             ratio_sign=length(unique(
             kinGroupRes[kinGroupRes$ratio_padj < 0.05, "family"]
             )),
             kinship_sign=length(unique(
             kinGroupRes[kinGroupRes$kinship_padj < 0.05, "family"]
             ))
             )
resTab
##   total_families ratio_sign kinship_sign
## 1             41          4            6
```

The most significant kinship group identified by the kinship group test is shown in the figure below. The mother (individual 17609) of the nuclear family representing this group and all her daughters have cancer (see figure below). This mother is however not directly related to the affected founder of this family, individual 17517, but did marry her son (id 17530; see figure above for the full pedigree of this family 432).

We are also submitting the familial incidence rate values calculated above with argument `label1` which are then displayed below the ID of each individual in the plot.

```
plotPed(kinGroup, id=kinGroupRes[kinGroupRes$family == "432", "group_id"][1],
    prune=TRUE, label1=fr)
```

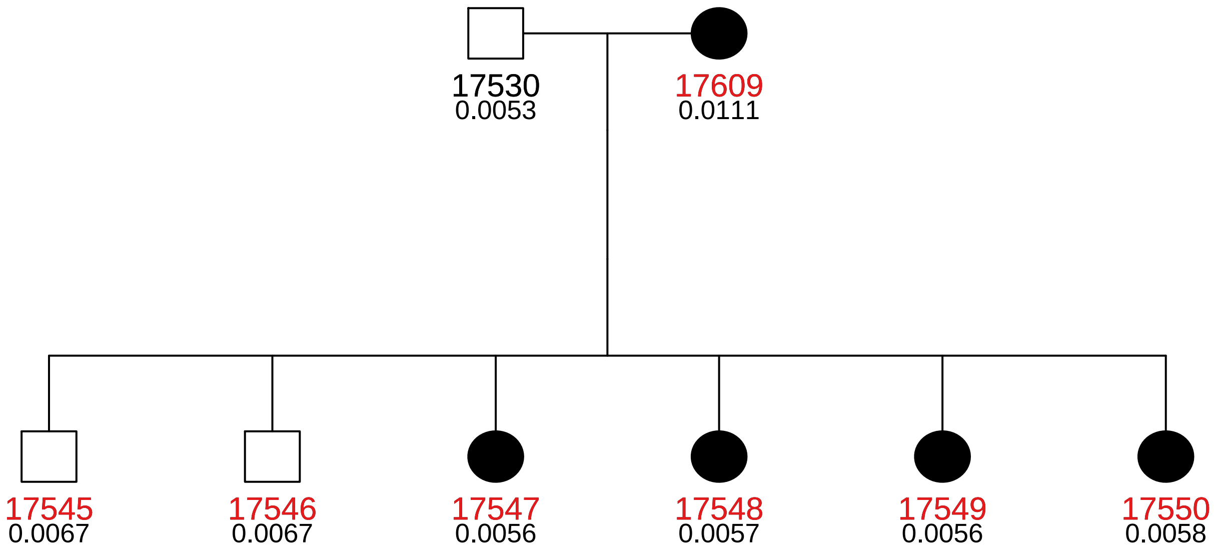

4.5 Exact inference for family disease clusters (*probability test*)

In this section, we apply the *probability test* that is based on the method from Yu et al [6] for inference on family disease clusters. We use the corresponding implementation in the gap package, that, due to an implementation specific limitation, can however not be directly applied to large pedigrees as the present, but requires definitions of smaller pedigree sub-sets (cliques).

We will thus restrict the analysis to families with fewer than 22 individuals and provide this information with the argument `cliques`. Unfortunately, this excludes most families in the present pedigree that exhibit significant familial aggregation of cancer.

```
## First we load the trait/affected information into the FAData object.
trait(fad) <- tcancer

## Next we determine the number of phenotyped individuals per family.
famAff <- pedigree(fad)[, c("family", "affected")]
## Exclude individuals that were not phenotyped.
famAff <- famAff[!is.na(famAff$affected), ]
## Calculate the number of phenotyped per family.
famSize <- table(famAff$family)

keepFams <- names(famSize)[famSize < 22]

## Extract the family and restrict to those on which we can perform the analysis.
famCliq <- fad$family
famCliq <- famCliq[famCliq %in% keepFams]
```

Now we can run the analysis.

```
probRes <- probabilityTest(fad, trait=tcancer, traitName="Cancer",
               cliques=famCliq, nsim=nsim)
probResTab <- result(probRes)
head(probResTab)
```

```
##    trait_name total_phenotyped total_affected phenotyped affected group_id
## 22     Cancer             2202            154         87        5       22
## 32     Cancer             2202            154         87        5       32
## 33     Cancer             2202            154         87        5       33
## 49     Cancer             2202            154         87        5       49
## 7      Cancer             2202            154         87        5        7
##    family group_phenotyped group_affected pvalue  padj
## 22     22               20              1  0.898 0.898
## 32     32               17              1  0.898 0.898
## 33     33               19              1  0.898 0.898
## 49     49               19              1  0.898 0.898
## 7       7               12              1  0.898 0.898
```

We plot the pedigree for the most significant clique. Notice however, that there are no significant results, as it can be seen from the `pvalue` and `padj` columns in the result table above.

```
plotPed(probRes, id=probResTab[1, "group_id"])
```

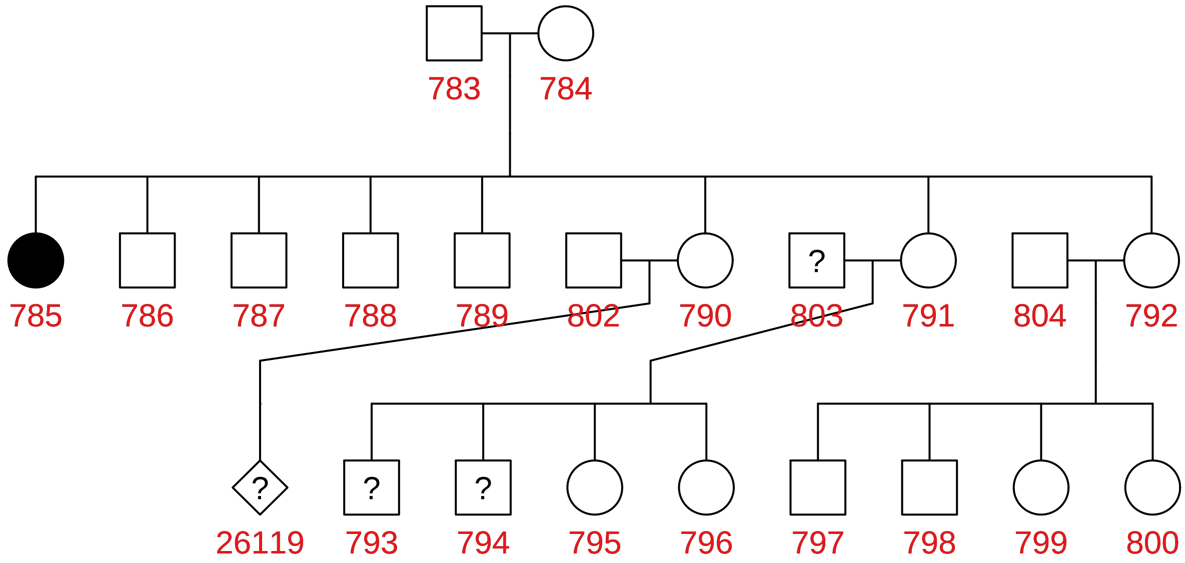

References

1. Sinnwell JP, Therneau TM, Schaid DJ: **The kinship2 R package for pedigree data.** *Human heredity* 2014, **78**:91–93.

2. Thiele H, Nuernberg P: **HaploPainter: A tool for drawing pedigrees with complex haplotypes.** *Bioinformatics* 2005, **21**:1730–1732.

3. Purcell S, Neale B, Todd-Brown K, Thomas L, Ferreira MAR, Bender D, Maller J, Sklar P, Bakker PIW de, Daly MJ, Sham PC: **PLINK: a tool set for whole-genome association and population-based linkage analyses.** *American journal of human genetics* 2007, **81**:559–575.

4. Hill JR: **A kinship survey of cancer in the Utah Mormon population**. In *Cancer incidence in defined populations. banbury report 4.* Edited by Cairns MJ, Lyon JL, Skolnic M. Cold Spring Harbor, NY; 1980:299–318.

5. Kerber RA: **Method for calculating risk associated with family history of a disease.** *Genetic epidemiology* 1995, **12**:291–301.

6. Yu C, Zelterman D: **Statistical inference for familial disease clusters.** *Biometrics* 2002, **58**:481–491.
